# Barriers and facilitators in cervical cancer screening uptake in Abidjan, Côte d'Ivoire in 2018: a cross-sectional study

**DOI:** 10.1186/s12885-021-08650-6

**Published:** 2021-08-25

**Authors:** Simon P. Boni, Franck Gnahatin, Jean-Claude Comoé, Boris Tchounga, Didier Ekouevi, Apollinaire Horo, Innocent Adoubi, Antoine Jaquet

**Affiliations:** 1 Programme National de Lutte contre le Cancer (PNLCa), Abidjan, Côte d’Ivoire; 2 Programme PAC-CI, Site ANRS Treichville, Abidjan, Côte d’Ivoire; 3 Jhpiego, a John Hopkins University affiliate, oncology department, Abidjan, Côte d’Ivoire; 4 Elizabeth Glaser Paediatric AIDS Foundation, Yaoundé, Cameroon; 5 University of Bordeaux, Inserm, French National Research Institute for Sustainable Development (IRD), UMR, 1219 Bordeaux, France; 6 Département de Santé Publique, Université de Lomé, Lomé, Togo; 7 Département mère-enfant, Université Félix Houphouët Boigny, Abidjan, Côte d’Ivoire; 8 Service de Gynéco-Obstétrique, Centre Hospitalier Universitaire de Yopougon, Abidjan, Côte d’Ivoire; 9 Service de cancérologie, Centre Hospitalier Universitaire de Treichville, Abidjan, Côte d’Ivoire; 10 Département Cancérologie-Immunologie-Hématologie, Université Félix Houphouët Boigny, Abidjan, Côte d’Ivoire

**Keywords:** Cervical cancer, Screening, Uptake, Côte d'Ivoire

## Abstract

**Backgrounds:**

Cervical cancer (CC) incidence remains unacceptably high in Côte d’Ivoire. In an effort to prevent this malignant condition, a national CC screening program has been scaled up in the country. This study aimed at assessing CC screening uptake and its associated factors in Abidjan in 2018.

**Methods:**

A cross-sectional survey was conducted from July to September 2018 in the main healthcare facilities of three randomly selected out of the eight health districts of Abidjan. During the study period, a standardized questionnaire was administrated by research assistants to all women aged 25 to 55 years old, attending the three participating facilities. Demographics, knowledge on CC, personal history of CC screening and reasons for not attending CC screening were collected. A logistic regression model was computed to document factors associated with reported CC screening uptake.

**Results:**

A total of 1158 women with a median age of 32 years (IQR [27–36]), including 364 (31.4%) with no formal education were included. Of those participants, 786 (67.9%) had ever heard about CC. CC screening uptake at least once was reported by 7.5% [95% CI: 6.0–9.0] participants. In multivariable analysis, being ≥45 years (aOR: 6.2 [2.3–17.2]), having a university level (aOR: 2.8 [1.2–6.6]) (versus non formal education) and access to mass campaign information (aOR: 18.2 [8.5–39.1]) were associated with a reported CC screening uptake. The main reported barriers to CC screening were unawareness towards CC screening (75.5%), negligence (20.5%), fear of CC detection (3.9%) and fear of additional costs (3.3%).

**Conclusion:**

CC screening uptake remains low despite current initiatives to support awareness and prevention in Abidjan. Awareness campaigns need to be massively increased with the adjunction of tailored messages based on the level of women’s education to enhance the CC screening coverage and reach the WHO goal of CC elimination by 2030.

## Background

Cervical cancer (CC) is the fourth most common cancer in women at a worldwide level with around 568,847 new cases and 311,365 deaths according the International Agency for Research on Cancer (IARC) [1]. Around 87% of the CC-related deaths occurred in low and middle income countries (LMICs) [2]. In 2018, approximately 1789 new cases and 1446 deaths were recorded in Côte d'Ivoire making this malignancy the second most common cancer and the leading cause of mortality by cancer in women [1].

The preponderant role of persistent oncogenic Human Papillomavirus Virus (HPV) infection and the subsequent long asymptomatic phase before invasive stages ensure evidence that a significant proportion of the CC-related burden could be prevented by HPV vaccine, early screening and precancerous lesions treatment [2]. In LMICs, as Pap smear alternative, World Health Organization (WHO) recommended the implementation of CC screening programs based on visual inspection (VI) or based on oncogenic HPV detection where available [3]. In the beginning of the last decade, some developing countries have successfully implemented pilot VI-based screening programs in HIV clinics before scaling up them outside HIV sites and towards their countries [4–6].

In Côte d'Ivoire, this program started in 2009 targeting women living with HIV (WLHIV) aged under 55 years, because of the association between HIV and precancerous lesions and CC [2]. This VI-screening program spread significantly by extension in reproductive health services in primary care and referral facilities in Abidjan and then in almost all health districts of the country. For women who were older, an invitation for a Pap smear test was proposed outside project sites as well as to women at the 6–8 post-natal visit by gynaecologists [7, 8].

Despite the efforts of the Ministry of Health (MoH) and civil society to increase access to CC screening, estimates of CC screening coverage was low (1.2% in the whole urban area of Abidjan) as demonstrated in a previous retrospective, population-based study in 2014 [7].

Since 2012, the program scaled up and all women aged 25 to 55 are invited to a screening each three years while WLHIV were sensitized for an annual screening in HIV clinics or HIV integrated facilities [9]. In 2019, according to the National Cancer Control Program (NCCP), over 450 trained healthcare workers mainly midwives were delivering CC screening through the “screen and treat” approach by performing cryotherapy the same day when applicable, irrespective of the women HIV status, in over 135 facilities, from primary and university health care level, throughout Côte d’Ivoire [7, 9].

According to the recent WHO call towards CC elimination by 2030, countries should ensure that at least 70% of their targeted population of women accessed to CC screening by 35, and again by 45 years of age [10]. Therefore, there is a need for evaluating the efforts from NCCP to prevent CC by updating data on CC screening uptake in primary and secondary health care facilities in Côte d'Ivoire. In addition, a better knowledge of factors influencing CC screening uptake could help in the definition of health priorities and lead to appropriate actions to improve CC screening programs in LMICs.

This study aimed at assessing CC screening uptake and its associated factors among women living in Abidjan, the economic capital of Côte d’Ivoire.

## Methods

### Study design

A cross-sectional study was performed from July to September 2018 in three health facilities of Abidjan, the economic capital of Côte d'Ivoire. Abidjan is a cosmopolitan and the most populous town of Côte d'Ivoire, with 4,707,404 inhabitants, representing a quarter of the overall population of the country [11].

### Setting and participants

Among the eight health districts of Abidjan, three were randomly selected. In each of these three districts, one healthcare facility from primary or secondary level was selected based on a short list of facilities where the sanitary area was high and the frequentation of population was optimal. Three facilities (“general hospital of Bingerville”, “general hospital of Adjamé” and “Urban community-based health facility of Yopougon-Andokoi”) were then conveniently selected. Of note, General hospitals are secondary level health facilities with health workers trained to perform various health services including surgical pathologies management while the urban community-based facilities deliver mainly primary care services.

During the study period, all women aged 25 to 55 years old, attending the selected healthcare facility were approached to participate in the survey, regardless of the reason for attendance. Each day, a list of eligible women was created at the desk entrance and allowed to record all women eligible to the survey.

### Data collection

A standardized questionnaire was adapted from a previous form, administered to WLHIV for the same purpose [12]. This standardized questionnaire was administrated to each participant in a dedicated office by trained healthcare workers (medical doctors and midwives) to collect sociodemographic characteristics including age, educational level, marital status, monthly income and possession of communication-related equipment, knowledge of CC and its risk factors, symptoms and prevention tools. In addition, the history of CC screening was also assessed as well as the barriers of CC screening by collecting reasons for no. Participants who previously heard about CC were asked some questions including the origin and clarity of information on CC, knowledge of CC symptoms, prevention methods and risk factors. A CC screening uptake was defined by having ever screened at least once in a lifetime regardless of the screening method (cytology, visual inspection or others).

### Data collection and statistical analysis

Data were captured in a dedicated database created under Epidata 3.1 and exported to STATA 14.0 (*StataCorp, College Station, Texas*) for statistical analysis. Numerical variables were described as medians and interquartile range (IQR) while categorical variables were described as frequencies with percentages and compared using Chi square test and Fisher exact when appropriate. Results with a *p* value < 0.05 were considered statistically significant. A logistic regression model was used to determine factors associated with CC screening uptake among women in general population in Abidjan through a step-wise backward procedure. As first step, variables associated with CC screening uptake with a *p*-value ≤0.20 were included in the full model. Then, variables that were not statistically associated with CC screening uptake and did not add any significant prediction to the model were subsequently removed. A < 0.05 was retained for statistical significance in final multivariable model.. Association between CC screening uptake and explanatory variables were estimated using adjusted odds ratios (aOR) with their associated 95% confidence intervals (IC).

### Ethical consideration

The present research has been performed in accordance with the Declaration of Helsinki and all methods were performed in accordance with the relevant guidelines and regulations. The study did not require approval from the national ethics committee of Côte d’Ivoire for the following reasons: first, the research did not include any planned intervention by the researcher; second, the women targeted by the research did not experience an invasion of privacy; third, no dissemination of the research findings would identify specific individuals. Based on the national ethics committee notice, the Ministry of Health provided administrative clearance to use and analyze data collected through its NCCP. This study was a part of a wide evaluation program of the CC screening initiatives in Côte d'Ivoire, which was authorized by a letter from the NCCP. Written informed consent was obtained from each of participant before the administration of the questionnaire. Participants confidentiality and privacy were ensured through anonymous questionnaire form. An ID code number was attributed to each participant.

## Results

### Sociodemographic characteristics

Overall, during the study period, 1172 eligible women attending the participating facilities were asked to participate in the study. For lack of time, 14 (1.2%) declined. Finally, 1158 women were enrolled with a median age of 32 years (IQR: [29–42]). Among them, 648 (56%) had primary or no education level, 764 (66%) were married or living with a sexual partner. In regards to monthly income, 651 (56.2%) of participants had less than the minimum wage in Côte d'Ivoire (60,000 XOF = ~ 120 USD). In addition, 1099 (94.9%) participants owned a television and 1110 (95.9%) at least one mobile phone. Radio and internet access through smartphone were owned by 896 (77.4%) and 379 (32.7%) participants, respectively. Table 1.

**Table 1:** Demographic characteristics of women stratified by CC screening uptake in Abidjan, Côte d’Ivoire, (*N* = 1158)

Sociodemographic characteristics	Total ***N*** = 1158		CC screening uptake ***N*** = 87		No CC screening uptake ***N*** = 1071		P value
	n (%)		n (%)		n (%)		
**Age,** years median (IQR*)	32 [27–36]		37	[30–43]	32	[26–38]	–
≤ 35	854	(73.7)	39	(44.8)	815	(76.1)	< 0.001
> 35	304	(26.3)	48	(55.2)	256	(23.9)	
**Marital status**							
Single	394	(34.0)	26	(29.9)	368	(34.4)	0.40
Living with a partner	764	(66.0)	61	(70.1)	703	(65.6)	
**Monthly income (USD**^α^**)**							
≤ 40 or none	110	(9.5)	8	(9.2)	102	(9.5)	< 0.001
40–160	639	(55.2)	25	(28.7)	614	(57.3)	
≥ 160	409	(35.3)	54	(62.1)	355	(33.2)	
**Educational level**							
No formal	364	(31.4)	9	(10.3)	355	(33.1)	< 0.001
Primary or Secondary	561	(48.5)	42	(48.3)	519	(48.5)	
University	233	(20.1)	36	(41.4)	197	(18.4)	
**Possession of communication equipment**							
Television	1099	(94.9)	86	(98.9)	1013	(94.6)	0.22
Radio	896	(77.4)	72	(82.8)	824	(76.9)	0.71
At least one mobile phone	1110	(95.9)	84	(96.6)	1026	(95.8)	0.89
Internet connexion through smartphone	379	(32.7)	51	(58.6)	328	(30.6)	< 0.001

*IQR: Inter Quartile Range; ^**α**^***USD: United States Dollars***

### Knowledge on cervical cancer screening

Among the participants, 786 (67.9%) had ever heard about CC, higher in women with university level (96.1%, *n* = 224) compared to primary/secondary (68.6%, *n* = 385) or no formal (48.6%, *n* = 177) education (*p* < 0.001). Their main source of information towards CC was medias (61.2%, *n* = 481), relatives (20.7%, *n* = 163), hospital through healthcare workers (14.5%, *n* = 114) and mass campaigns (7.3%, *n* = 57). Among the 786 who ever heard about CC, information on CC was not considered as clear for 672 (85.5%) participants. Table 2*.*

**Table 2:** Knowledge and attitudes towards cervical cancer stratified by education level among women who have ever heard about cervical cancer in Abidjan, Côte d’Ivoire, (*N* = 786)

	Total (***N*** = 786)		No formal education (***N*** = 177)		Primary/ Secondary (***N*** = 385)		University (***N*** = 224)		P value
	N (%)		n (%)		n (%)		n (%)		
Place where ever heard about CC^*^									
In an hospital	114	(14.5)	15	(8.5)	58	(15.1)	41	(18.3)	0.021
Heard about CC^*^ in the media	481	(61.2)	103	(58.2)	236	(61.3)	142	(63.4)	0.378
Heard about CC^*^ during campaigns	57	(7.3)	10	(5.7)	28	(7.3)	19	(8.5)	0.575
Heard about CC^*^ through relatives^μ^	163	(20.7)	53	(29.9)	73	(19.0)	37	(16.5)	0.007
Clarity of received CC* information									
Not clear / I did not understand	672	(85.5)	163	(92.1)	338	(87.8)	171	(76.3)	< 0.001
Very Clear / I understand it	114	(14.5)	14	(7.9)	47	(12.2)	53	(23.7)	
Symptoms of CC^*^are									
Genital bleeding	184	(23.4)	27	(15.3)	87	(22.6)	70	(31.3)	0.004
Leucorrhoea	110	(14.0)	21	(11.9)	45	(11.7)	44	(19.6)	< 0.001
Abdominal Pain	123	(15.7)	16	(9.0)	46	(12.0)	61	(27.2)	< 0.001
CC^*^is a preventable									
Yes	469	(59.7)	84	(47.5)	221	(57.4)	164	(73.2)	< 0.001
No	161	(20.5)	43	(24.3)	88	(22.9)	30	(13.4)	
Means of prevention for CC^*^(*N* **= 469)**									
Screening	385	(82.1)	55	(65.5)	182	(82.4)	148	(90.2)	< 0.001
Vaccine	151	(32.2)	21	(25.0)	77	(34.8)	53	(32.3)	< 0.001
Risk factors for CC^*^									
HIV infection	91	(11.6)	7	(4.0)	31	(8.1)	53	(23.7)	< 0.001
Multiple sexual partners	194	(24.7)	27	(15.3)	78	(20.3)	89	(39.7)	< 0.001
Early sexual initiation	185	(23.5)	22	(12.4)	74	(19.2)	89	(39.7)	< 0.001
**Reasons for no CC screening uptake (*N*** **= 699)**									
Unawareness towards CC screening	528	(75.5)	143	(85.6)	266	(77.3)	119	(63.3)	< 0.001
Negligence	143	(20.5)	15	(9.0)	71	(20.6)	57	(30.3)	< 0.001
Fear of being diagnosed cancer	27	(3.9)	4	(2.4)	18	(5.2)	5	(2.7)	0.212
Fear of additional costs	23	(3.3)	5	(3.0)	6	(1.7)	12	(6.4)	0.016
Fear of a bad reception	2	(0.3)	–	–	1	(0.3)	1	(0.5)	0.53

*CC: cervical cancer;^*μ*^*relatives: parents, friends or living partner;****∞****Respondents had the possibility to choose over one choice;*^***£***^*only respondents for each variable among participants who ever heard about CC were considered, missing category was not specified; Except for the “total column”, proportions represent the number of women for each modality out of all women in the education level category*

In addition, CC was known as a preventable disease for 469 (59.7%) participants, higher in University (73.2%, *n* = 164) compared to primary/secondary (57.4%, *n* = 221) or no formal education (47.5%, *n* = 84) groups (*p* < 0.001). Regarding CC risk factors, HIV infection, multiple sexual partners and early sexual initiation were known as contributing to CC for 91 (11.6%), 194 (24.7%) and 185 (23.5%) participants, respectively.

### Cervical cancer screening uptake

Overall, the proportion of CC screening uptake was 7.5% [95% CI: 6.0–9.0]. Among the 87 who had ever been screened for CC, 45 (51.7%) and 42 (48.3%) were screened through VI and pap smear, respectively. A reported CC screening was completed following mass awareness campaigns and after recommendation from a healthcare worker through 6–8 post-delivery visit for 38 (43.6%) and 36 (41.4%) women, respectively. A self-initiative was reported by 13 (14.9%) women.

### Reasons for no CC screening uptake

Among the 699 participants with no history of CC screening, reasons of not attending screening were lack of sufficient knowledge on CC (75.5%, *n* = 528), carelessness or negligence (20.5%, *n* = 143), fear of cancer (3.9%, *n* = 27), fear of additional costs (3.3%, *n* = 23) and the fear of a bad reception at CC screening facility (0.3%, *n* = 2). Carelessness (230.3%) was common among women with university level (*p* < 0.001) while the lack of sufficient knowledge on CC (85.6%) were the highest reported reason in no formal education one else (*p* < 0.001).

### Associated factors with cervical cancer screening uptake

In multivariate analysis, being ≥45 years (aOR: 6.2 [2.3–17.2]), having a university level versus no formal education (aOR: 2.8 [1.2–6.6]), receiving an information on CC considered as clear (aOR: 2.6 [1.9–3.4]) and access to mass campaign information (aOR: 18.2 [8.5–39.1]) were associated with CC screening uptake. Table 3.

**Table 3:** Factors associated with CC screening uptake among women previously aware of CC in Abidjan, Côte d’Ivoire, 2018 (*N* = 786)

Variables	CCS^**¥**^ uptake		Multivariable analysis (initial model)		Multivariable analysis (final model)	
	n/N*	%	OR (CI 95%)	p value	aOR (CI 95%)	p value
**Age (years)**						
≤ 30	21/383	5.5	1	–	1	–
[30–45[	56/359	15.6	2.5 (1.4–4.7)	0.003	3.0 (1.6–5.5)	< 0.001
≥ 45	10/44	22.7	5.1 (1.8–14.3)	0.002	6.2 (2.3–17.2)	< 0.001
**Marital status**						
Single	27/291	9.3	1	–	–	–
Living with a partner	60/495	12.1	1.4 (0.8–2.6)	0.216	–	–
**Educational level**						
No formal	10/177	5.7	1	–	–	–
Primary or Secondary	41/385	10.7	1.9 (0.8–4.3)	0.142	1.9 (0.9–4.2)	0.126
University	36/224	16.1	2.5 (1.0–6.0)	0.045	2.8 (1.2–6.6)	0.017
Monthly income (^α^USD)						
≤ 40 USD	8/69	11.6	1			
40–160	25/397	6.3	1.0 (0.4–2.6)	0.964	–	–
≥ 160	54/320	16.9	0.7 (0.4–1.3)	0.213	–	–
**Source of information on CC**^**¥**^						
Through medias	25/529	4.7	1		–	–
In an hospital	22/87	25.3	6.1 (2.9–12.5)	< 0.001	5.8 (2.8–11.8)	< 0.001
Through relatives^£^	17/115	14.8	2.6 (1.2–5.3)	0.012	2.7 (1.3–5.6)	0.008
During a mass campaign	23/55	41.8	17.6 (8.1–37.9)	< 0.001	18.2 (8.5–39.1)	< 0.001
**Clarity of information**						
Not clear for me	42/672	6.3	1	–	1	–
Very clear for me	45/114	39.5	2.3 (1.7–3.1)	< 0.001	2.6 (1.9–3.4)	< 0.001
**Knowing CC**^**¥**^**as a preventable disease**						
**vNo**	15/317	4.7	1		–	–
**Yes**	72/469	15.4	1.8 (0.9–3.6)	0.072	–	–

^**¥**^***CCS:*** Cervical Cancer Screening

*n/N: number of women who had ever been screened for CC/Number of women per category

^**£**^**Relatives:** friends or parents; ^**α**^***USD:*** United States Dollars

## Discussion

In this study, we report a low CC screening uptake among women attending primary and secondary healthcare facilities in the urban area of Abidjan, Côte d'Ivoire. Access to a comprehensive information about CC and being reached through mass awareness campaign were identified as major determinants in uptake to CC screening. In addition, the most important barrier to CC screening uptake reported by women was the lack of sufficient knowledge about CC, far behind economic considerations.

Estimated uptake of CC screening is lower than previous studies conducted in Nigeria, Ethiopia, Kenya and Cameroon which reported rates ranging from 19.6 to 43.5% [13–16]. A similar finding of 7.8% was reported from Tanzania while the lowest rate was reported from Zimbabwe (3.8%) [17, 18]. The low access to screening services among women in our population could be partly explained by their relatively young age. Indeed, the young profile of our population study could explain partly this low rate compared to participants from Cameroon and Ethiopia where the median age was 41 and 36 years, respectively. Furthermore, the significant association between older age especially in peri-menopausal women and CC screening uptake found in this study rightfully point out the interest for screening gained as the age progresses. Though this survey reported a low awareness regarding CC and its prevention method, the higher estimate of CC screening uptake found in this study compared with the CC screening coverage of 1.2% reported previously in a five-years population-based survey (2010–2014). This potential increase in CC screening uptake should encourage NCCP leaders to pursue their efforts [7].

In all cases, this study demonstrated a limited access to CC screening services in Abidjan by women from general population compared to their HIV-infected counterparts. In 2017, a previous cross-sectional survey in Abidjan revealed that 96.1%WLHIV on antiretroviral treatment had heard about CC yielding a greater CC screening uptake with nearly two-third (59.7%) ever being screened [12]. This significantly higher CC screening uptake was likely related to early sensitization and screening pilot project in HIV clinics. Despite the scale up of CC screening, there seems to be an unmet need in the coverage of the target population for whom those current mass communication campaigns were designed for as defined by national authorities. Though older women and WLHIV are both highly vulnerable population to CC, younger women also need to be reached with those messages, especially for prevention purposes. CC being among the most preventable forms of cancer, integrating CC screening habits into a lifestyle at a young age would guarantee early detection of potential cancerous lesions. Additional efforts in targeting younger women should be done by NCCP and program partners in the perspective of prevention of CC.

That involves an aggressive communication in communities and a systematic proposition for screening to all women seeking a hospital, regardless the purpose of the visit.

This study revealed that the comprehension of the messages on CC is a determinant of the decision to undergo screening. Women who estimated being well informed had positive attitudes towards screening compared to those who were ill-informed. There is an urgent need for improving clear information about CC among communities by tailoring messages according to cultural habits and age-groups behaviors. A recent meta-analysis underlined the positive impact of culturally tailored education interventions on raising attendance for screening [19]. These messages should include necessarily key points such as (i) the serious concern that represents CC and its preventable aspect (can be cured if diagnosed early), (ii) the possibility to prevent it through regular screenings and complete immunization by HPV vaccine and (iii) the safety and effectiveness of prevention methods for CC mortality reduction [20]. In this perspective, the MoH must ensure that healthcare workers and peer-communities have adequate skills and knowledge on CC and their ability to communicate these key messages. The success of the cervical cancer screening program is based upon solid training of all stakeholders involved in mass awareness campaigns across the country.

Despite growing efforts to scale-up systematic CC screening in Côte d'Ivoire, it still remains mainly opportunistic at the population level and there is no organised program inviting women to regular screening with incentives and reminder support. Meanwhile, mass campaign information related to CC screening are multiplying and relayed by various media supports. This translates into a growing number of women being aware of CC screening but still a limited effective access to CC screening. The present study was not designed to formally assess the precise impact of mass CC screening programs. However, in our study, being informed on CC screening through mass campaign was strongly associated with CC screening uptake supporting the usefulness of this communication process. Generally, during mass awareness campaigns, sensitization and often screening are offered free of charge, unlike in healthcare facilities routinely delivering CC screening where at least 5 USD are required for a VI test. These activities, mainly conducted by NGOs are preceded by mass and social media promotion yielding an increase in women participation.

They are more and more regular in the context of Côte d'Ivoire, with recently a frank involvement of religious and administrative leaders. Despite the scale up of CC prevention, these findings inform future efforts in terms of strengthening CC screening services utilization by increasing mass awareness campaign in both peri-urban and rural settings. In fact, poor conditions in which populations are living impact the success of prevention strategies, as illustrated in Burkina Faso [21] and Nigeria [22]. Therefore, strategies aiming at bringing CC screening closer to women by mitigating geographic and financial barriers in LMICs should be encouraged. Global mobilization, added with the pursuit of a structured and continuous program of creation of proximity sites, should be the key to achieve the ambitious 70% coverage, set by the MoH. Civil society initiatives, community mobilization through more and effectiveness engagement of their leaders should resulted in increasing of CC screening awareness and CC screening uptake. In fact, women in general population should be more exposed to CC screening, through both hospital-based and community-based strategies. That requires a better organization and an empowerment of all NGOs engaged in CC control and a close collaboration with the NCCP for a better optimization of CC information penetration and to guarantee an equitable access to screening services across all the districts of the country. Besides, this whole organization must be evaluated by reinforcing monitoring activities, initiated at national level through the District Health Information Software (DHIS2) only recently, and therefore be more useful for policy makers. In fact, because of funding constraints, regular monitoring activities through aggregated data and key performance indicators are lacking in Côte d'Ivoire. As sustained CC screening initiatives expand in the country, the NCCP should list these monitoring activities as a key priority, while addressing the need to standardize indicators on CC prevention along with care continuum, from all primary and secondary healthcare facilities, regardless their HIV management status-clinics or integrated.

## Limitations

Our study has some limitations despite the useful information provided. First, the use of CC screening services was self-reported exposing to desirability bias. No verification has been done despite the required details on the visit for CC screening (date, professional status of provider and facility). However, this study used a structured questionnaire previously validated and tested in WLHIV for assessment of CC screening uptake [12]. Second, this study was conducted among women attending a limited number of healthcare facilities from public sector, in the capital of the country because of organisational aspects and financial procedures which did not allow the execution of the initial plan. The findings from this study may not be representative of women attending the private sector and form the whole country including rural settings where CC uptake could be the lowest because of socioeconomic inequities and geographical barriers to CC screening. Nevertheless, a multistage sampling procedure and a rigorous participant selection in facilities from two different levels of the health system were applied to limit potential selection bias.

## Conclusion

The CC screening uptake remains low in the context of constraining-resources and low literacy rate settings such as Abidjan, despite the commitment of the health authorities. Mass awareness campaigns need to be increased with the adjunction of tailored messages based on the level of women’s education to enhance the CC screening coverage. As efforts are undertaken by the NCCP to promote a wider and sustainable access to CC screening in the country, monitoring activities are also one of the key priorities that need to be reinforced to evaluate the impact of this preventive strategy at a population level through standardized aggregated indicators.

## Data Availability

The datasets generated and analyzed from this study are not publicly available as they contain confidential information that could compromise privacy. As appropriated, they could be made available with non-identifiable aspects from the corresponding author on reasonable request.

## Abbreviations

HPVs: Human papillomaviruses
LMICs: Low and middle income countries.
AOR: Adjusted Odd’s Ratio
CC: Cervical cancer
FIGO: International Federation of Gynaecology and Obstetrics
MoH: Ministry of health
NCCP: National Cancer Control Program
WHO: World Health Organization
WLHIV: Women living with HIV
